# i-MoMCARE: AI-enabled mobile app for maternal and child health care in Cambodia – a pilot implementation and evaluation study

**DOI:** 10.1136/bmjhci-2025-101691

**Published:** 2026-04-24

**Authors:** Hendra Goh, Dyna Khuon, Mengieng Ung, Sreymom Oy, Chhavarath Dary, Chhorlika Khim, Sveng Chea Ath Chhay, Yan Fang Lee, Rattana Kim, Siyan Yi, Vonthanak Saphonn

**Affiliations:** 1 Saw Swee Hock School of Public Health, National University of Singapore, Singapore; 2 University of Health Sciences, Phnom Penh, Cambodia; 3 SingHealth Duke-NUS Global Health Institute, Singapore; 4 National Maternal and Child Health Center, Phnom Penh, Cambodia; 5 Singapore Institute of Technology, Singapore

**Keywords:** Delivery of Health Care, Health Services Research

## Abstract

**Objectives:**

In Cambodia, village health support groups (VHSGs) are central to maternal and child health (MCH) service delivery, yet maternal mortality remains high with regional disparities. Although digital health solutions show promise, their real-world use is underexplored. This study therefore aims to evaluate the i-MoMCARE app in supporting VHSGs and health centre staff for delivering MCH services.

**Methods:**

A mixed-methods approach guided by the Reach, Effectiveness, Adoption, Implementation and Maintenance (RE-AIM) framework was used. Reach was assessed using programme records (number of users trained and MCH cases managed). Effectiveness and adoption were evaluated using the mHealth App Usability Questionnaire (MAUQ) and qualitative data on user experience and intention to use. Implementation was examined through system usage monitoring and feedback on workflow integration. Maintenance was explored using qualitative insights on long-term use, barriers and recommendations.

**Results:**

44 VHSGs and 10 health centre staff were trained, and 96 MCH cases were managed with the app. Usefulness was rated highly (MAUQ 5.58, SD 1.04), with users appreciating the user-centric design that improved usability and integration into daily workflows. Effectiveness was similarly high (MAUQ 5.50, SD 0.95); the clinical decision support system and point-of-care alerts were valued as reliable decision-support tools, and the electronic medical record (EMR) was seen as a secure data repository. However, adoption was hindered by hardware and software limitations (MAUQ 5.79, SD 0.75), including device availability and low digital literacy.

**Discussion:**

Our findings highlight the value of the i-MoMCARE app in supporting VHSGs, particularly those with limited training, by enhancing their decision-making. The study also lays the groundwork for a unified EMR in primary care. However, concerns around hardware and software limitations hinder adoption and sustained use.

**Conclusion:**

To enable scale-up, we recommend reducing access barriers and supporting sustained use through diversifying the platform’s design and implementing phased incentivisation strategies.

**WHAT IS ALREADY KNOWN ON THIS TOPIC**

- While maternal mortality in Cambodia has substantially reduced over the past decades, maternal and child health (MCH) remains a critical challenge, especially in rural communities where access to quality services is limited.
- Globally, digital health solutions—particularly mobile health (mHealth) applications—have shown promise in strengthening health systems by improving service delivery, information flow and patient engagement.
- Despite this momentum, there is limited empirical evidence on the feasibility, adoption and sustainability of mHealth technologies within Cambodia’s health system.
- This gap highlights the need for implementation research to inform effective integration of digital health solutions into routine MCH services in Cambodia.

**WHAT THIS STUDY ADDS**

- The i-MoMCARE app offered a digital alternative to traditional paper-based systems, featuring a user-friendly interface that supported high usability and acceptance among frontline health workers.
- Key features—such as the clinical decision support system and point-of-care medical alerts—were widely regarded as useful aids in guiding clinical decisions, particularly in settings with limited supervision or high uncertainty.
- However, hardware- and software-related challenges further constrained the intention to adopt and sustained usage.

**HOW THIS STUDY MIGHT AFFECT RESEARCH, PRACTICE OR POLICY**

- The i-MoMCARE app demonstrated how digital tools can enhance clinical decision-making, streamline data collection and support frontline health workers in low-resource settings.
- However, successful scale-up and sustained implementation require addressing persistent infrastructural and capacity-related challenges.
- Targeted investments in infrastructure, training and user support are essential to fully realise the potential of mHealth solutions like the i-MoMCARE app in strengthening primary healthcare.

## Introduction

 Maternal and child health (MCH) remains a critical global health priority, particularly in low- and middle-income countries (LMICs) such as Cambodia.^1^ However, substantial disparities persist, with rural areas continuing to record significantly higher mortality rates than urban regions.^2^ These inequities stem from enduring challenges such as limited access to healthcare services, inadequate infrastructure and persistent health workforce shortages in remote communities.^3 4^

In this context, village health support groups (VHSGs)—community health workers (CHWs) recruited from local populations—play a pivotal role in delivering essential MCH services, especially in rural areas where over 75% of the population resides.^5^ Their responsibilities span various paediatric services,^6 7^ pregnancy care^8^ and communicable and non-communicable disease prevention.^9 10^ However, despite their vital presence, challenges such as high maternal mortality ratios and stark regional disparities persist in remote provinces, underscoring the need for innovative solutions to strengthen MCH outcomes.^11^

Over the years, digital health solutions (DHS) have been increasingly recognised for improving MCH services in resource-limited settings.^12^,^15^ mHealth apps, in particular, have shown strong potential in supporting community-based healthcare delivery. For instance, in Bangladesh, the mCARE app enhanced pregnancy surveillance and health-seeking behaviours, with a modelling study estimating over 3000 lives saved in 8 years.^16^ Similarly, Indonesia’s mPosyandu app helped CHWs identify and address child nutrition issues early.^17^ These successes illustrate how digitalisation can strengthen the effectiveness of community-based health programmes—an outcome highly relevant to Cambodia’s health system.

In this context, the study team developed the i-MoMCARE app in March 2024 to support VHSGs and health centre staff in rural Cambodia. At its core, the app integrates key features, including a clinical decision support system (CDSS), point-of-care medical alerts, intelligent referral mechanisms and scheduling tools, to support MCH service delivery. It also includes educational resources to strengthen staff capacity and an electronic medical record (EMR) system to streamline data management. By addressing the operational needs of VHSGs and the systemic challenges of Cambodia’s healthcare system,^18^ the i-MoMCARE app represents a promising solution.

Despite robust evidence that mHealth apps improve MCH service delivery, their practical relevance and sustained user engagement remain underexplored.^19^ Challenges, such as unfamiliarity with digital tool use, non-intuitive interfaces and infrastructural limitations, often undermine the intention to use consistently.^20 21^ Moreover, mHealth apps that fail to align with end-user needs risk being abandoned in favour of traditional paper-based systems, suggesting a mismatch between DHS and on-the-ground operational realities.^20^ These gaps underscore the importance of prioritising user-centred design and longitudinal evaluations to ensure that DHS are practical and sustainable for their intended users.

Therefore, this study aims to evaluate the i-MoMCARE app. By examining the potential and limitations of this intervention, the research seeks to contribute to the broader understanding of how DHS can be effectively integrated into community-based healthcare systems in Cambodia.

## Methods

### Study design

This multiphase mixed-methods implementation study comprised a cross-sectional survey and qualitative interviews with VHSGs, health centre staff and pregnant women. The intervention was developed over three phases.

#### Phase 1: co-design workshop

An iterative co-design workshop was conducted over 3 months, guided by the Sanders and Stappers co-design framework.^22^ Key stakeholders collaboratively translated end-user needs identified in prior formative research into app features.^23 24^ Proposed features were prioritised using a feasibility–impact approach, resulting in seven core features for initial development. Two versions of the system were developed: a mobile app for VHSGs and a web-based platform for health centre staff. Prototype testing with end users informed final refinements prior to deployment (see online supplemental file 1).

#### Phase 2: training workshop

A 2-day training workshop was delivered to VHSGs and health centre staff from two health facilities. The training combined refresher sessions on MCH content with hands-on digital literacy training tailored to each platform. Participants practised using the system through guided case scenarios, supported by printed user guides.

#### Phase 3: pilot study

A 3-month pilot study (June–August 2024) was conducted in Battambang Province to evaluate the feasibility, acceptability and preliminary effectiveness of the i-MoMCARE app among VHSGs and health centre staff. Feasibility was assessed based on system usage rates, data completeness and participant retention; acceptability was evaluated through user satisfaction and perceptions, and preliminary effectiveness was explored through perceived improvements in service delivery. A pragmatic sampling approach was adopted, whereby all available VHSGs and health centre staff within the intervention sites (n=54) were included to ensure representation of end-users at both community and facility levels. All VHSGs and health centre staff were asked to use the system for household registration, clinical decision support and health education during routine service delivery. System use was monitored, and ongoing support was provided to encourage engagement. At the end of the pilot, all VHSGs and health centre staff were invited to complete the survey questionnaire. Purposive sampling was subsequently employed to capture diverse perspectives across user groups (VHSGs, health centre staff and pregnant mothers), ensuring variation in experience and context during in-depth interviews.

Additional details on phases 1–3 are provided in the online supplemental file 2.

### Data collection

Recruitment of participants was conducted collaboratively by the core research team members from the National University of Singapore (MU and SO) and the University of Health Sciences, Cambodia (CD and DK). Invitations were extended to potential VHSGs and health centre staff through formal channels within the provincial health department. Initial contact was made via email or phone call, facilitated through coordination with the respective health centre directors who served as gatekeepers to ensure appropriate communication while maintaining voluntariness.

#### Cross-sectional surveys

After 3 months, all VHSGs and health centre staff completed a usability evaluation tool—the mHealth App Usability Questionnaire (MAUQ) for standalone apps.^25^ MAUQ is widely recognised as a benchmark for assessing the usability of mHealth apps.^26^ This validated 18-item questionnaire evaluates usability across three key domains—ease of use, usefulness and satisfaction. VHSGs and health centre staff were asked to rate each item on a seven-point Likert scale, ranging from 1 (strongly disagree) to 7 (strongly agree), with higher overall and domain-specific scores indicating greater app usability. Domain scores were calculated as the mean of all items within each domain.

#### Qualitative interviews

VHSGs and health centre staff were purposively recruited based on their age and years of working experience to participate in in-depth interviews (IDIs). Meanwhile, pregnant mothers were recruited for focus group discussions (FGDs) during their outpatient visits to the health centres. An interview guide was developed based on the relevant literature^12 27 28^ and pilot tested to ensure clarity. The guide included questions about user perceptions of the i-MoMCARE app, perceptions of specific features and their impacts and recommendations for app improvement. Trained qualitative researchers (DK, MU and SO) conducted all interviews in person at the health centres. All interviews were audio-recorded and transcribed verbatim. The duration of interviews ranged from 20 to 55 min. The study followed the Consolidated Criteria for Reporting Qualitative Research (COREQ) checklist^29^ to ensure methodological rigour and transparency (online supplemental file 3).

### Data analysis

#### Quantitative data

MAUQ scores were summarised using appropriate descriptive statistics such as mean and SD. All statistical analyses were conducted using R V.4.2.4.

#### Qualitative data

Qualitative data from IDIs and FGDs were transcribed verbatim. Six steps of thematic analysis, as suggested by Braun and Clarke,^30^ were undertaken to explore users’ perceptions of the i-MoMCARE app, its associated features and recommendations for improvement. Two analysts (HG and YFL) reviewed the transcripts independently and coded the transcripts using NVivo 15. Any discrepancies in coding were resolved through discussion with senior team members (SY).

#### Data integration

Integration occurred at both the analysis and interpretation stages. Quantitative and qualitative findings were first analysed separately, followed by data triangulation through a side-by-side comparison to identify convergence, divergence and complementarity between datasets. Quantitative results provided measurable indicators of usability, while qualitative insights contextualised these findings by explaining user experiences, perceptions and implementation challenges.

#### Intervention evaluation

The intervention’s evaluation was guided by the RE-AIM framework, in which each domain (Reach, Effectiveness, Adoption, Implementation and Maintenance) was assessed individually using predefined indicators outlined in online supplemental file 4.

## Results

### Reach

#### Phase 1: co-design workshop

A total of 16 stakeholders participated in this phase, including five representatives from the Ministry of Health and National Maternal and Child Health Centre, six researchers and five software developers. The stakeholders had diverse expertise in medicine, midwifery, pharmacy, public health, data science and engineering.

#### Phase 2: training workshop

All 56 invited participants (46 VHSGs and 10 health centre staff) attended the training, achieving a 100% participation rate. However, by the end of the training, two VHSGs withdrew from the study, citing challenges with mobile phone use. Table 1 presents the characteristics of 54 participants in the training. Most health centre staff and VHSGs were female, with a mean age of 38.7 (SD 10.5) years. While all health centre staff completed tertiary education, all VHSGs had high school education or below.

**Table 1:** Characteristics of participants

Characteristic	Pregnant mothers (n=96) phase 3: pilot study (total maternal and child health cases seen)	VHSGs and HC staff (n=54) phase 2: training workshop phase 3: pilot study (cross-sectional survey)	VHSGs and HC staff (n=14) phase 3: pilot study (in-depth interview)	Pregnant mothers (n=14) phase 3: pilot study (focus group discussion)
Age (years)				
Mean±SD	28.6±7.5	38.7±10.5	43.1±9.9	30.3±6.1
Gender, n (%)				
Female	96 (100)	40 (74.1)	8 (57.1)	14 (100)
Male	0 (0)	14 (25.9)	6 (42.9)	0 (0)
Education, n (%)				
Primary	57 (59.4)	10 (18.5)	1 (7.1)	5 (35.7)
Secondary/High School	36 (37.5)	34 (63.0)	9 (64.3)	9 (64.3)
Tertiary	3 (3.1)	10 (18.5)	4 (28.6)	
Profession, n (%)				
HC staff		10 (18.5)	4 (28.6)	
VHSG		44 (81.5)	10 (71.4)	
Number of children, n (%)				
One child	31 (32.3)			8 (57.1)
Two children	50 (52.1)			4 (28.6)
Three children	15 (15.6)			2 (14.3)

HC, health centre; VHSG, village health support group.

#### Phase 3: pilot study

During the pilot study, 800 cases were registered by VHSGs, and 96 MCH cases were documented in the EMR. The mean age of the mothers was 28.6 years. Over half (59.4%) had attained only primary education, and 52.1% reported having two children. The services were delivered by 41 VHSGs and five health centre staff using the i-MoMCARE app. Three VHSGs (6.8%) discontinued using the app, citing a lack of interest. Five health centre staff (50%) did not log in using their assigned user IDs. However, follow-up discussions revealed that staff resorted to account sharing due to a shortage of available computers to avoid the inconvenience of repeatedly logging in and out. While the exact number of health centre staff who independently used the i-MoMCARE app remains unclear, this posed a limitation in tracking actual system use. Table 1 summarises the characteristics of the three engaged groups. First, it presents characteristics of 96 mothers whose information was captured through the app. Second, it describes the 54 health centre staff and VHSGs who participated in the cross-sectional survey. Lastly, it documents the characteristics of 14 health centre staff and VHSGs who participated in the IDIs, along with 14 pregnant women who later took part in FGDs.

### Effectiveness

#### Usability of the i-MoMCARE app

Most participants highlighted that the i-MoMCARE app’s user-centric design enhanced usability, making it intuitive and suitable for daily use (table 2). The ‘simple and familiar layout’ was frequently noted as a key facilitator in ease of use. Users appreciated how the app resembled a traditional paper-based documentation system. This familiarity encouraged continued usage, with one VHSG expressing confidence in adopting the system long-term. Beyond its ease of navigation, the app’s usability also extended to service recipients. Several mothers emphasised the app’s ‘well-scoped design’, effectively supporting the full spectrum of MCH. The broad coverage ensured the app’s practical applicability and relevance, motivating users to integrate it into daily practice. Quantitatively, the i-MoMCARE app received an average MAUQ score of 5.50 (SD 0.95) for ease of use and interface, indicating high usability. This convergence showed the i-MoMCARE app’s user-friendly design, enabling seamless use in daily workflows.

**Table 2:** Joint display of user perception towards the i-MoMCARE app (usability)

Overarching themes	Qualitative findings	Quantitative item	Mean (SD)	Median (Min, Max)	Meta inference
User-centred design facilitates usability	User friendly layout enhances familiarity and encourages sustained usage *‘(Layout is simple) and easy to use. If now the organisation asks us to continue using it, I will keep using it*’. (VHSG, F, 33) *‘Also, the system is very similar to the books we currently use to record patient information, so it is a good move as we work to digitalise our primary healthcare system’.* (HC staff, M, 31) Well-scoped design to daily work renders greater practical applicability *‘I think the features developed are logical, covering the whole spectrum of a woman from not pregnant (family planning services) to pregnant (ANC services, giving birth (delivery services), post-delivery recovery (PNC care) and even services for the newborn (child services). It is really a one-stop service for mothers and children!’* (Pregnant mother, F, 29) *‘I like the application as it can be used daily for our community. I think it is (well-scoped too)to contain the whole trajectory of conception to child care’.* (HC staff, F, 27)	**Overall ease of use and interface score**.	5.50 (0.95)	6 (1, 7)	The quantitative data consistently reflects high ratings for ease of use and interface design, aligning with qualitative insights that emphasise the app’s intuitive layout and its resemblance to existing documentation systems. This convergence reinforces i-MoMCARE’s user-friendly design, enabling seamless usability in daily workflows.
		The app was easy to use.	5.54 (0.82)	6 (3, 6)	
		It was easy for me to learn to use the app.	5.26 (1.18)	6 (1, 7)	
		The navigation was consistent when moving between screens.	5.41 (1.02)	6 (2, 7)	
		The interface of the app allowed me to use all the functions (such as setting a step goal, logging a physical activity session, receiving notifications) offered by the app.	5.41 (1.07)	6 (2, 7)	
		Whenever I made a mistake using the app, I could recover easily and quickly.	5.54 (0.91)	6 (3, 7)	
		I like the interface of the app.	5.67 (0.85)	6 (2, 7)	
		The information in the app was well organised, so I could easily find the information I needed.	5.67 (0.82)	6 (3, 7)	

ANC, antenatal care; F, female; HC, health centre; M, male; PNC, postnatal care; VHSG, village health support group.

#### Usefulness of the i-MoMCARE app

Participants highlighted the usefulness of the i-MoMCARE app in improving clinical confidence, work efficiency, interprofessional collaboration and data accuracy through automation (table 3). These qualitative insights were supported by a high MAUQ score of 5.58 (SD 1.04), underscoring the app’s practical value in clinical settings. Specifically, VHSGs reported increased confidence in delivering patient care due to the CDSS’s structured guidance. Before using the app, some users were ‘unsure’ about what to look out for during patient visits. However, the CDSS offered reassurance by reinforcing decision-making, particularly for frontline workers with limited formal training.

**Table 3:** Joint display of user perception towards the i-MoMCARE app (usefulness)

Overarching themes	Qualitative findings	Quantitative item	Mean (SD)	Median (Min, Max)	Meta inference
Usefulness on patient care and clinical practice	Enhancing clinical confidence through AI-assisted decision-making *‘When I started using the app, I knew more information especially regarding patients who are at risk. Before that, I was unsure of what to look out for’.* (VHSG, F, 42) *‘As* (*the workflow) is guided, I am sure that I provide the correct care. At times, I am unsure of what care to give during the visit, but now with the app, I feel more confident’.* (VHSG, M, 52) Improving work efficiency and interprofessional collaboration *‘Moreover, as data are now digitalised, it will provide an up-to-date patient record which both the VHSGs and health centre staff can view. Last time, I noticed that VHSGs always had to bring the books back to the health centre for the staff to review before my visit, so it is kinda troublesome’.* (Pregnant mother, F, 25) Automated processes reduce human error and eliminate laborious tasks *‘Also, it saves us time from having to record data manually as everything is electronically now. It may reduce errors too, for example if the clinical parameters are recorded wrongly, error messages will be generated and we cannot submit it. I think it is good as it ensures data accuracy’.* (HC staff, F, 29)	Overall usefulness score.	5.58 (1.04)	6 (1, 7)	The high quantitative ratings for usefulness in clinical practice and patient health management align with qualitative insights, which highlight how i-MoMCARE enhances clinical confidence, streamlines workflow efficiency and fosters interprofessional collaboration.
		The app would be useful for my healthcare practice.	5.70 (0.96)	6 (3, 7)	
		The app improved my access to delivering healthcare services.	5.85 (0.68)	6 (4,7)	
		The app helped me manage my patients’ health effectively.	5.63 (1.07)	6 (1,7)	
		This app has all the functions and capabilities I expected it to have.	5.74 (0.94)	6 (2,7)	
		This mHealth app provides an acceptable way to deliver healthcare services, such as accessing educational materials, tracking my own activities, and performing self-assessment.	5.65 (1.10)	6 (2,7)	
		I could use the app even when the Internet connection was poor or not available.	4.93 (1.48)	6 (2, 7)	

F, female; HC, health centre; M, male; VHSG, village health support group.

Furthermore, the transition from paper-based to digital patient records was seen as an improvement in work efficiency. A pregnant mother observed that previously, it was ‘troublesome’ for VHSGs because they had to transport patient records manually. With the app, real-time data synchronisation allowed staff to instantly access and update patient information, reducing delays and improving interprofessional collaboration. Lastly, participants valued automation features for reducing manual workload and ensuring data accuracy. The app eliminated the need for manual record-keeping and report generation, significantly reducing errors. Built-in validation mechanisms and medical alerts also helped to flag incorrect clinical parameters, preventing submission of erroneous data.

### Adoption

#### Satisfaction and intention to adopt the i-MoMCARE app

While quantitative findings indicate high satisfaction and adoption intent, as reflected in the MAUQ score of 5.79 (SD 0.75), qualitative insights revealed a more complex reality (table 4). Despite a positive reception, the limited workforce readiness, driven by infrastructure constraints, posed challenges to sustaining adoption. For instance, insufficient devices and poor internet coverage made it difficult for users to access the system. Beyond infrastructure, digital literacy gaps among users, particularly senior staff, were cited as another barrier to adoption. Some staff lacked basic computer and smartphone proficiency, making transitioning from paper-based systems to a digital platform difficult. Additionally, even among users who had undergone digital training, partial understanding of the system persisted, leading to concerns about operational confidence in using the app.

**Table 4:** Joint display of user perception towards the i-MoMCARE app (adoption)

Overarching themes	Qualitative findings	Quantitative item	Mean (SD)	Median (Min, Max)	Meta inference
Limited readiness for digital transition hinders widespread adoption	Technical barriers comprising of hardware and software limitations reduce adoption intent *‘Currently, we have only two computers but ANC, PNC, delivery and child services are all done in different consultation rooms. So, it is difficult to use the app as we do not have enough computers for all staff’.* (HC staff, M, 31) *‘From my knowledge, some health centres in other provinces have no internet connection as the cell site is too far away. So, for them, it might be difficult to use the app. I think before that, we need to ensure that the internet coverage can reach there’.* (HC staff, F, 27) Poor digital literacy prolongs learning and adoption of new technology *‘Also, senior staff might face difficulties having to adapt to electronic systems as some of them don’t even know how to operate a computer, so it may be a potential barrier when you want to roll out nationwide’.* (HC staff, M, 31) *‘I haven't fully understood it 100%, but 70% is understandable. There is still concern about using it as I’m not fully proficient’. (*VHSG, F, 42)	Overall satisfaction score.	5.79 (0.75)	6 (3, 7)	While quantitative findings indicate high satisfaction and intention to adopt i-MoMCARE, qualitative insights suggest a more complex reality. Workforce readiness remains a challenge due to limited digital tools and gaps in digital literacy, potentially hindering sustained adoption.
		The app adequately acknowledged and provided information to let me know the progress of my action.	5.78 (0.63)	6 (4, 7)	
		I feel comfortable using this app in social settings.	5.67 (0.99)	6 (2, 7)	
		The amount of time involved in using this app has been fitting for me.	5.81 (0.75)	6 (3, 7)	
		I would use this app again.	5.89 (0.57)	6 (4, 7)	
		Overall, I am satisfied with this app.	5.81 (0.83)	6 (3, 7)	

ANC, antenatal care; F, female; HC, health centre; M, male; PNC, postnatal care; VHSG, village health support group.

### Implementation

#### Programme delivery and consistency

Implementing the study intervention followed a structured approach aligned with the published protocol.^31^ The app was deployed in accordance with the planned framework, ensuring that all key features, training sessions, and workflows were delivered as designed across all participating health centres. Beyond procedural adherence, programme fidelity was also monitored through the training workshops and supervision logs. Trainers assessed whether end users correctly followed the data entry procedures, referral protocols and communication workflows outlined during training.

### Maintenance

Several recommendations were offered to improve the long-term maintenance of the i-MoMCARE app (table 5). These suggestions focused on leveraging extrinsic motivation to drive long-term use and on improving app infrastructure to enhance functionality. One salient theme identified was that resistance to digital transition could slow adoption and reduce intention to use. To overcome this, participants suggested initial incentives to lower the barrier to entry. Once users had experienced the app’s tangible benefits, continued use became more likely even without incentives. In addition, to further bolster uptake, participants emphasised the need for enhancing the interoperability, as the app is only currently compatible with Android devices. Expanding compatibility to tablets and other operating systems could help to overcome computer shortages and improve portability, allowing more users to use the app flexibly.

**Table 5:** Recommendations for improving adoption and maintenance

Themes	Subthemes	Illustrative codes
Enhancing app infrastructure to improve functionality	Secure inpatient communication network	*‘We can create a text-message feature* (*in this app) so the health centre staff can contact and ask us questions regarding the patients. Vice-versa, we can also communicate securely with health centre staff on patient conditions during home visits’.* (VHSG, M, 41)
	Presence of live technical assistance	*‘Sometimes the app has errors or bugs, and it can be really frustrating—especially when a patient is waiting, and we can’t get it to work. It can get quite embarrassing when it takes too long to fix. That’s why I really hope we can text the IT to bring up such issues quickly’.* (HC staff, F, 29)
	Strengthening interoperability to lower barrier of entry	*‘Currently, the app is not Apple compatible as it is currently only designed for Android phones. Also, it is not compatible with tablets either. It is better if we make it tablet compatible so we can overcome the shortage in computers by using tablets. It may also be more portable too and we can bring it around during patient consultation’.* (HC staff, F, 44)
Leveraging extrinsic motivation to drive and maintain long-term use	Incentivisation to encourage early adoption	*‘I think monthly remuneration will help to encourage some of the VHSGs to start using it. As you know, some people are resistant to new changes, so you need to encourage them. After they realised the usefulness of this app, even when you don’t reward them, they will not revert back to pen and paper’*. (HC staff, F, 32)
	Diversified training modalities for sustaining use	*‘To keep the training more sustainable, we can create videos to teach them step-by-step how to use the app. So, whenever they forget, they can just rewatch the videos and they will continue using it without trouble’.* (VHSG, F, 42) *‘Also, we can have yearly refresher course to inform us on the latest updates and also take the opportunity to introduce the technology to new VHSGs’.* (VHSG, M, 52)

F, female; HC staff, health centre staff; M, male; VHSG, village health support group.

## Discussion

This study offers a comprehensive evaluation of the practical relevance and sustained engagement of the i-MoMCARE app from the end-users’ perspective. Notably, 54 VHSGs and health centre staff were trained. 800 patients were registered in the i-MoMCARE app during the study period, including 96 MCH patients who were actively enrolled and followed up via the web version. Concerning effectiveness, end-users perceived the i-MoMCARE app as user-friendly and highly useful, particularly through targeted features. While participants expressed a desire to adopt the app, challenges remain in maintaining long-term usage. Overall, within the RE-AIM framework, the intervention demonstrated moderate reach, positive perceived effectiveness and high usability, but adoption and maintenance were constrained by hardware limitations, digital literacy challenges and infrastructure gaps.

In terms of effectiveness, the i-MoMCARE app offers several valuable features. Among the most well-received is the built-in CDSS. As highlighted in the literature, pregnant women frequently present with multiple comorbidities, increasing the complexity of clinical management and the cognitive demands associated with prescribing decisions.^32^ Our findings suggest that the technological support for clinical workflow was widely perceived as a reliable knowledge resource supporting clinical decision-making across various stages of pregnancy. Functioning as a protocol navigator, it was perceived to guide users through delivering consistent, guideline-aligned care while instilling greater prescribing confidence.^33^ This promotes the standardisation of quality of care and reduces cognitive burden among healthcare providers, allowing them to refocus on patient-centred care.

Complications associated with high-risk pregnancies also place a considerable burden on the public healthcare system.^34^ The i-MoMCARE app includes a point-of-care medical alert feature for detecting danger signs. By leveraging a predefined algorithm, the system analyses clinical parameters to estimate the likelihood of pregnancy- and child-related complications. Participants viewed these alerts as meaningfully impacting patient care, particularly by enabling early clinician–patient communication and intelligent referrals to tertiary care. This finding is similar to other research indicating that risk prediction tools support shared decision-making and facilitate timely interventions.^35 36^ When effectively implemented, such predictive features may enable more proactive management of high-risk pregnancies and potentially contribute to reducing MCH-related healthcare costs.^37 38^

Another hallmark of the i-MoMCARE app is the introduction of an EMR system for health centres. While EMRs are currently available in some tertiary care settings in Cambodia, their implementation remains fragmented.^38^ Meanwhile, the primary healthcare system continues to rely on traditional paper-based documentation. Our findings highlight several limitations of handwritten records, including potential breaches of patient confidentiality and challenges with timely information exchange. For example, data booklets may be misplaced or damaged due to natural wear and tear, and delays in VHSGs delivering records to health centre staff before patient visits can compromise patient safety and disrupt continuity of care. The newly implemented EMR serves as a secure data estate, is perceived to improve interoperability and might facilitate real-time information sharing among healthcare providers.^39^ Furthermore, it streamlines clinical documentation by enabling users to create ambient notes, edit live data and generate discharge summaries within a user-friendly interface, significantly reducing administrative burnout.^40^ While the EMR is currently limited to managing MCH cases, it establishes a scalable foundation for developing a national, unified EMR system across Cambodia’s healthcare system.

Despite the perceived benefits of the i-MoMCARE app, participants in our study expressed concerns regarding its long-term adoption. Findings indicate that hardware-related challenges could impede sustained use. In our pilot study, about 50% of health centre staff did not log in with their assigned IDs due to device shortages, raising concerns over equitable adoption and the limited reach of the DHS. While the most direct solution lies in broader investment in the digital infrastructure of the health system, such efforts are often impractical in many LMICs, where sustainable health financing remains a persistent challenge.^41 42^ As an alternative, it is paramount to diversify DHS design to ensure interoperability with widely available or more affordable devices, such as tablets, wearables and other operating systems. This approach may mitigate infrastructure limitations to promote broader adoption across resource-constrained settings.

Concurrently, software-related challenges—remarkably low levels of digital literacy and resistance—were reported to diminish users’ confidence and willingness to adopt the technology. A phased incentivisation scheme is therefore recommended to reduce resistance to change. In the initial implementation phase, users may receive financial or non-financial incentives to provide temporary support and compensate for the additional effort required to adapt to DHS.^43^ Once users recognise the practical benefits, a negotiated transition can be introduced, where incentives are gradually withdrawn. At this stage, users are more likely to view the platform as a helpful job aid rather than an added burden, facilitating continued use without external reinforcement. This approach supports a shift from extrinsic to intrinsic motivation, fostering long-term adoption and maintenance into routine practice.^44^

### Limitations

Due to funding constraints and the scope of the research objectives, the app was limited to MCH services. However, health centres themselves provided broader care, requiring users to switch between digital and paper systems, reducing usage. Although 800 cases were registered, only 96 cases were managed with the web version at the health centres. This posed as a limitation in tracking actual system use. Also, given the short pilot timeframe, the RE-AIM domains of *Effectiveness* and *Maintenance* were only partially assessed. Future studies should include longitudinal follow-up and clinical indicators to assess health impact and sustainability.

## Conclusion

This study evaluated i-MoMCARE’s relevance and engagement in MCH delivery. Findings highlight the perceived usefulness of CDSS in supporting VHSGs and the potential of EMR for primary care digitalisation. However, adoption and maintenance were limited by hardware and software constraints, suggesting the need for improved interoperability and phased incentivisation strategies to enhance uptake.

## Supplementary material

10.1136/bmjhci-2025-101691 online supplemental file 1

10.1136/bmjhci-2025-101691 online supplemental file 2

10.1136/bmjhci-2025-101691 online supplemental file 3

10.1136/bmjhci-2025-101691 online supplemental file 4

## Data Availability

Data are available upon reasonable request.
