# Supplementary material for: i-MoMCARE: AI-enabled mobile app for maternal and child health care in Cambodia – a pilot implementation and evaluation study
Source: BMJ Health Care Inform. 2026 Apr 24;33(1):e101691. doi: 10.1136/bmjhci-2025-101691 (PMC13110616; doi:10.1136/bmjhci-2025-101691)
Supplement: online supplemental file 1 [file bmjhci-33-1-s001.pdf]

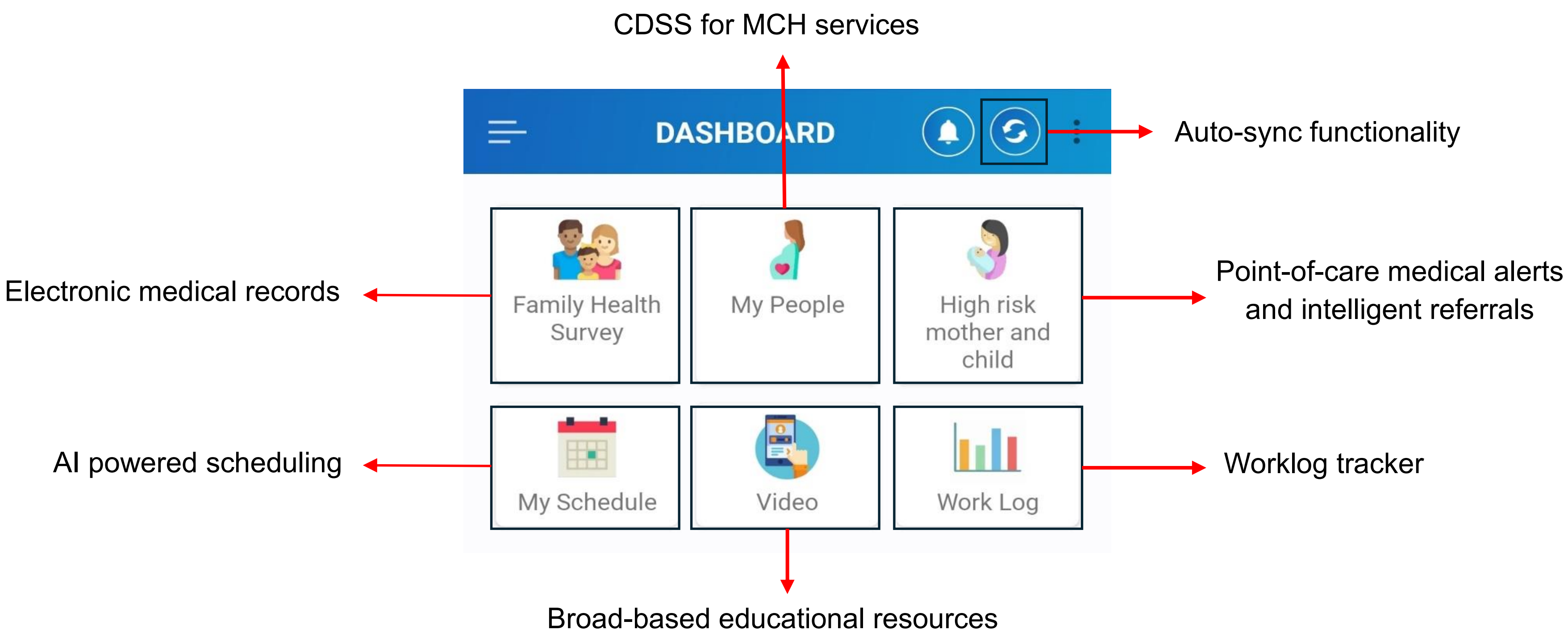

Fig 1. Snapshot of i-MoMCARE (app version) for VHSGs

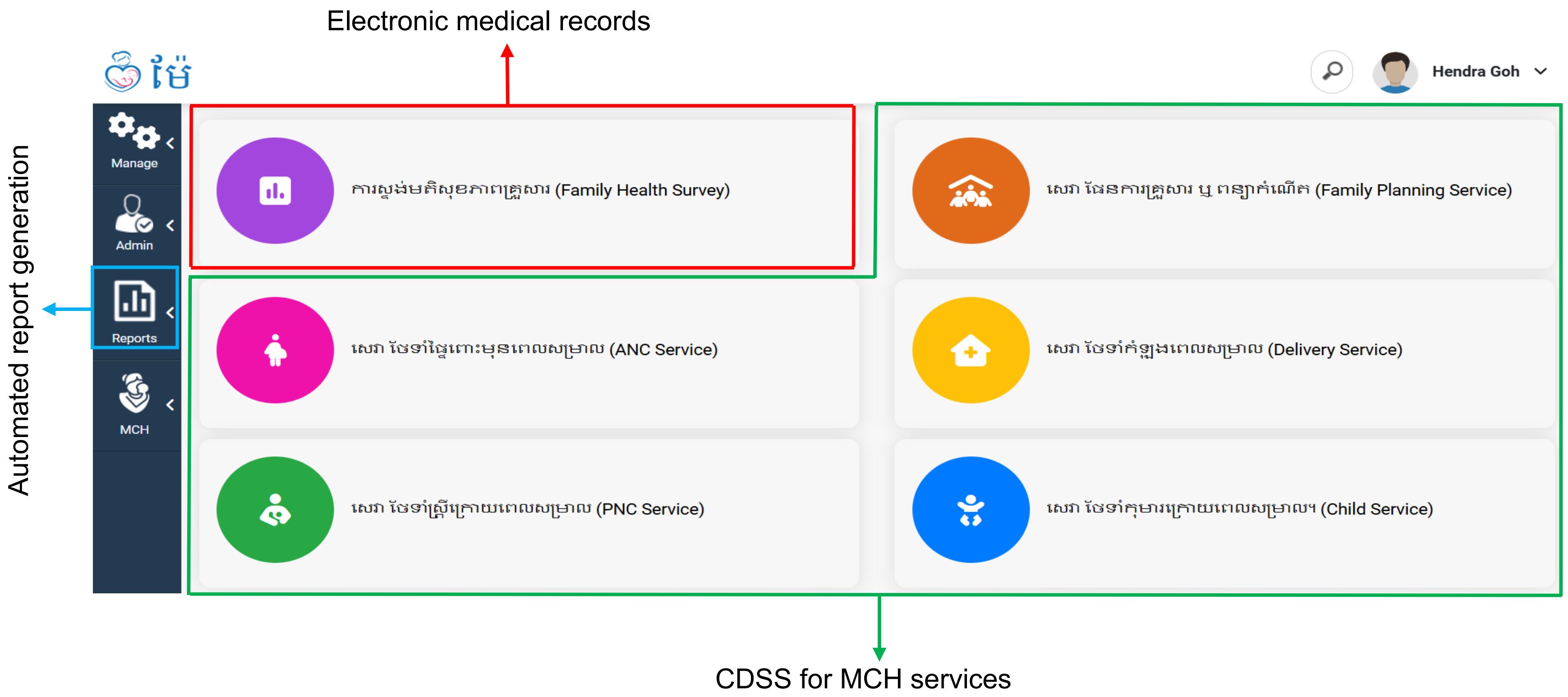

Fig 2. Snapshot of i-MoMCARE (web version) for health centre staff
