# Supplementary material for: i-MoMCARE: AI-enabled mobile app for maternal and child health care in Cambodia – a pilot implementation and evaluation study
Source: BMJ Health Care Inform. 2026 Apr 24;33(1):e101691. doi: 10.1136/bmjhci-2025-101691 (PMC13110616; doi:10.1136/bmjhci-2025-101691)
Supplement: online supplemental file 2 [file bmjhci-33-1-s002.docx]

**Supplementary File 2. Additional details on methodology Phases 1-3**

*Phase 1: Co-design workshop*
In Phase 1, an iterative co-design workshop took place over three months. The co-design approach developed by Sanders & Stappers was undertaken to guide the design of the digital app (14). Stakeholders included representatives from the Ministry of Health (MoH), National Maternal and Child Health Centre (NMCHC), the University of Health Sciences (UHS), and the software development team. In this phase, stakeholders and researchers from the study team collaboratively proposed app features for initial development. To guide the development, stakeholders first strategically translated end-user needs—previously identified through formative research (15, 16)—into actionable app features. Subsequently, a feasibility–impact approach was adopted during collaborative discussions, in which each proposed app feature was assessed and ranked based on its potential impact on MCH outcomes, relevance to identified user needs, and feasibility of implementation within existing project resources and time constraints. The seven highest-ranked app features were therefore prioritised for initial development.

Based on these consultations, the software development team implemented the proposed features into the i-MoMCARE app. Two versions of the i-MoMCARE app were available: an app-based version developed for VHSGs and a web-based version designed for health centre staff. Supplementary File 1 shows a snapshot of the i-MoMCARE app and web version. In the final phase of the workshop, ten end users (four VHSGs and six health centre staff) participated in the initial prototype test, providing feedback to inform refinements before the app was released to users.

*Phase 2: Training workshop*
Phase 2 involved a two-day training workshop for VHSGs and health centre staff from two designated facilities in Battambang province. The training was structured into two key components. The first focused on a refresher course on MCH topics, reinforcing essential knowledge for service delivery. The second component consisted of digital literacy training, equipping VHSGs with skills to use the i-MoMCARE app, while health centre staff were trained on the web version. The training was followed by a 20-minute free-and-easy session, during which VHSGs and health centre staff could explore the app and the web independently. Subsequently, the trainers presented various patient case scenarios and asked the participants to use the app or web version to simulate care provision and medical record entry. Printed lecture notes and user guides were distributed as reference materials. All attendees received USD 5 reimbursement for participation.

Fidelity was also monitored through the training workshops and supervision logs. Trainers assessed whether end users correctly followed the data entry procedures, referral protocols, and communication workflows outlined during training. Although no formal quantitative fidelity assessment was conducted due to the short pilot duration, qualitative feedback from field observations and participant interviews provided valuable insights into process consistency and quality of delivery. These observations highlighted areas for improvement, such as the need for regular refresher training and real-time troubleshooting support, which informed future large-scale implementation and evaluation of the app.

*Phase 3: Pilot study*
Phase 3 consisted of a three-month pilot study (June–August 2024) designed to evaluate the feasibility, acceptability, and preliminary effectiveness of the i-MoMCARE app among VHSGs and health centre staff. Feasibility was assessed based on system usage rates, data completeness, and participant retention; acceptability was evaluated through user satisfaction and perceptions; and preliminary effectiveness was explored perceived improvements in service delivery.

All available VHSGs and health centre staff from the two intervention sites (n=54) were included using a pragmatic sampling approach to ensure representativeness of both community and facility-based healthcare providers. Minimal exposure thresholds were defined as at least one patient interaction recorded in the system per user per month. System use was continuously monitored through backend analytics capturing frequency of logins, data entries, and feature utilisation.

During the study, VHSGs and health centre staff were trained to register patients through the EMR, follow the CDSS workflow during consultations, and use the multimedia resources for community health education. Monthly remuneration (USD 5 for VHSGs and USD 15 for health centre staff) was provided as an incentive to encourage consistent engagement. At the conclusion of the pilot study, several VHSGs and health centre staff were purposively invited to complete a cross-sectional survey and in-depth interviews (IDIs) for evaluation purposes. Meanwhile, pregnant women were invited to participate in focus group discussions (FGDs) to share their experiences as service recipients.
