# Supplementary material for: i-MoMCARE: AI-enabled mobile app for maternal and child health care in Cambodia – a pilot implementation and evaluation study
Source: BMJ Health Care Inform. 2026 Apr 24;33(1):e101691. doi: 10.1136/bmjhci-2025-101691 (PMC13110616; doi:10.1136/bmjhci-2025-101691)
Supplement: online supplemental file 3 [file bmjhci-33-1-s003.doc]

**Consolidated criteria for reporting qualitative research (COREQ): 32-item checklist**

| **Item number** | **Guide questions** | **Reported on** |
| --- | --- | --- |
| **Domain 1: Research team and reﬂexivity** |  |  |
| *Personal Characteristics* |  |  |
| 1. Interviewer/facilitator | Which author/s conducted the interview or focus group? | Data collection in Methods section. |
| 2. Credentials | What were the researcher’s credentials? E.g. PhD, MD | MD, PhD, MPH. |
| 3. Occupation | What was their occupation at the time of the study? | Academic faculty in the University. |
| 4. Gender | Was the researcher male or female? | Males and females. |
| 5. Experience and training | What experience or training did the researcher have? | Training in health services and public health. |
| *Relationship with participants* |  |  |
| 6. Relationship established | Was a relationship established prior to study commencement? | No relationship was established prior to study commencement. |
| 7. Participant knowledge of the interviewer | What did the participants know about the researcher? e.g. personal goals, reasons for doing the research | Participants were aware of the primary purpose of the study (Data collection in Methods section). |
| 8. Interviewer characteristics | What characteristics were reported about the inter viewer/facilitator? e.g. Bias, assumptions, reasons and interests in the research topic | Interviewer has extensive experience in qualitative research (Data collection in Methods section). |
| **Domain 2: study design** |  |  |
| *Theoretical framework* |  |  |
| 9. Methodological orientation and Theory | What methodological orientation was stated to underpin the study? e.g. grounded theory, discourse analysis, ethnography, phenomenology, content analysis | Thematic analysis (Data analysis in Methods section). |
| *Participant selection* |  |  |
| 10. Sampling | How were participants selected? e.g. purposive, convenience, consecutive, snowball | Purposive sampling was employed (Data collection in Methods section). |
| 11. Method of approach | How were participants approached? e.g. face-to-face, telephone, mail, email | Email and phone call (Data collection in Methods section). |
| 12. Sample size | How many participants were in the study? | In Results section. |
| 13. Non-participation | How many people refused to participate or dropped out? Reasons? | In Results section. |
| *Setting* |  |  |
| 14. Setting of data collection | Where was the data collected? e.g. home, clinic, workplace | In health centres. |
| 15. Presence of non-participants | Was anyone else present besides the participants and researchers? | No one was present besides the participants and researchers. |
| 16. Description of sample | What are the important characteristics of the sample? e.g. demographic data, date | Characteristics of the sample were described (in Results, Table 2). |
| *Data collection* |  |  |
| 17. Interview guide | Were questions, prompts, guides provided by the authors? Was it pilot tested? | Interview guides were developed based on relevant literature. The guides were pre-tested to ensure clarity and accuracy (Data collection in Methods section). |
| 18. Repeat interviews | Were repeat interviews carried out? If yes, how many? | No repeat interviews were carried out. |
| 19. Audio/visual recording | Did the research use audio or visual recording to collect the data? | All interviews were audio-recorded following verbal consent (Data collection in Methods section). |
| 20. Field notes | Were ﬁeld notes made during and/or after the interview or focus group? | Field notes were written in every interview to capture insights (Data collection in Methods section). |
| 21. Duration | What was the duration of the inter views or focus group? | The duration of interviews ranged from 20 to 55 minutes (Data collection in Methods section) |
| 22. Data saturation | Was data saturation discussed? | Data saturation was reached after the 14th interview (Result section). |
| 23. Transcripts returned | Were transcripts returned to participants for comment and/or correction? | Transcripts were not returned to participants. |
| **Domain 3: analysis and ﬁndings** |  |  |
| *Data analysis* |  |  |
| 24. Number of data coders | How many data coders coded the data? | Two independent coders (Data analysis in Methods section). |
| 25. Description of the coding tree | Did authors provide a description of the coding tree? | The coding tree was developed through NVivo 15® but not presented in the manuscript. |
| 26. Derivation of themes | Were themes identiﬁed in advance or derived from the data? | Themes were derived from the data (Data analysis in Methods section). |
| 27. Software | What software, if applicable, was used to manage the data? | NVivo 15® used (Data analysis in Methods section). |
| 28. Participant checking | Did participants provide feedback on the ﬁndings? | Participants did not provide feedback on the findings. |
| *Reporting* |  |  |
| 29. Quotations presented | Were participant quotations presented to illustrate the themes/ﬁndings? Was each quotation identiﬁed? e.g. participant number | Quotations were presented and participant number provided (Table 3 and 4). |
| 30. Data and ﬁndings consistent | Was there consistency between the data presented and the ﬁndings? | Consistency was checked (in Results section). |
| 31. Clarity of major themes | Were major themes clearly presented in the ﬁndings? | Major themes were clearly presented (Table 3 and 4). |
| 32. Clarity of minor themes | Is there a description of diverse cases or discussion of minor themes? | Minors themes were described (in Results section). |
