# Supplementary material for: i-MoMCARE: AI-enabled mobile app for maternal and child health care in Cambodia – a pilot implementation and evaluation study
Source: BMJ Health Care Inform. 2026 Apr 24;33(1):e101691. doi: 10.1136/bmjhci-2025-101691 (PMC13110616; doi:10.1136/bmjhci-2025-101691)
Supplement: online supplemental file 4 [file bmjhci-33-1-s004.docx]

| Supplementary File Table 4. RE-AIM indicators and data collection details | | | | |
| --- | --- | --- | --- | --- |
| Domain | **Indicator** | **Engaged groups** | **Data sources** | **Timing** |
| Reach | Number and characteristics of stakeholders attended the Phase 1 Co-design workshop | Co-design stakeholders | Field notes | Post Phase 1 Co-design workshop |
|  | Number and characteristics of participants attended the Phase 2 Training workshop | VHSGs and health centre staff | Field notes | Post Phase 2 Training workshop |
|  | Number and characteristics of patients served during Phase 3 Pilot study | Pregnant mothers and children | Health centre data | Post Phase 3 Pilot study |
| Effectiveness | Usability of i-MoMCARE | VHSGs, health centre staff, pregnant mothers | Cross-sectional survey Qualitative interview | Post Phase 3 Pilot study |
|  | Usefulness of i-MoMCARE | VHSGs, health centre staff, pregnant mothers | Cross-sectional survey Qualitative interview | Post Phase 3 Pilot study |
| Adoption | Adoption intent and satisfaction towards i-MoMCARE | VHSGs, health centre staff, pregnant mothers | Cross-sectional survey Qualitative interview | Post Phase 3 Pilot study |
| Implementation | Description of program delivery and consistency | N/A | Field notes | Post Phase 3 Pilot study |
| Maintenance | Recommendations to improve adoption and maintenance | VHSGs and health centre staff | Qualitative interview | Post Phase 3 Pilot study |

Note: The RE-AIM framework used in this study was adapted to align with the intervention's implementation stage. Specifically, the Effectiveness dimension was measured using proximal indicators, such as usability and usefulness—early indicators of the app’s perceived effectiveness—rather than clinical effectiveness. Likewise, the Maintenance dimension was assessed based on end users’ expectations and strategies for ensuring continued engagement with the i-MoMCARE app. These modifications were necessary due to the early phase of implementation but should be taken into account when interpreting the findings.
